# Towards detailed predictions of coastal ecosystem function under climate change

**DOI:** 10.1371/journal.pbio.3002430

**Published:** 2023-12-12

**Authors:** Diego R. Barneche, Renato A. Morais

**Affiliations:** 1 Australian Institute of Marine Science, Crawley, Australia; 2 Oceans Institute, The University of Western Australia, Crawley, Australia; 3 Paris Sciences et Lettres Université, École Pratique des Hautes Études, EPHE-UPVD-CNRS, USR 3278 CRIOBE, Perpignan, France

## Abstract

The complex ways in which food availability and warming will restructure ecosystems remains poorly understood. This Primer explores a new simulation study in PLOS Biology suggesting that a 30% change in resource supply might cause more pervasive effects than a +2.5˚C warming effect.

## Teaser

The complex ways in which ongoing warming will restructure ecosystems remains poorly understood. A new simulation study in *PLOS Biology* suggests that expected changes in food resources for marine consumers will outpace the direct, pervasive effects of predicted +2.5°C warming.

**Fig 1:**
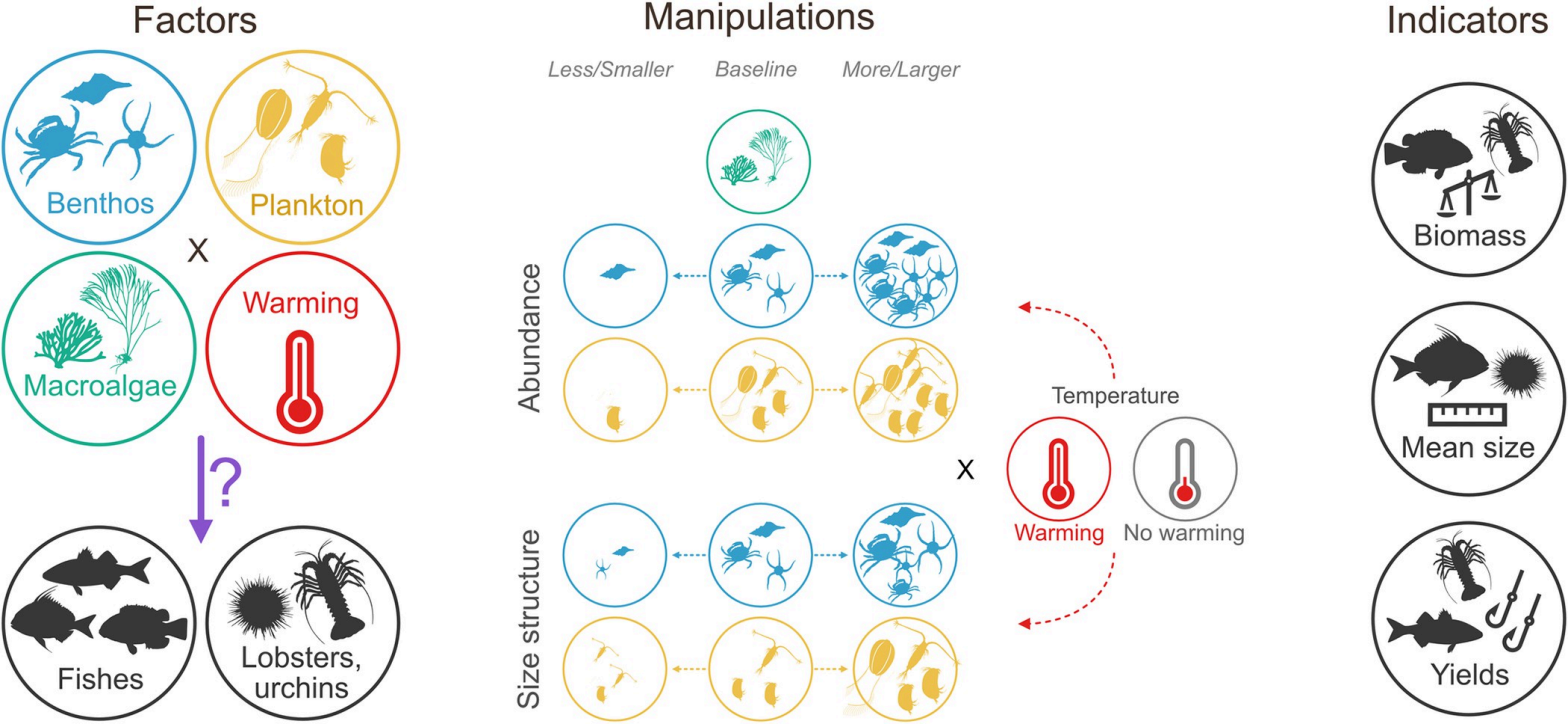
The simulation framework developed by Audzijonyte and colleagues [1]. See main text for description. All silhouettes have been downloaded from phylopic.org, under a “CC0 1.0 Universal Public Domain Dedication” license.

Coastal ecosystem shifts are unfolding in complex ways because of changes in ocean temperature. Although we understand what warming might do to siloed components of an ecosystem—for example, physiological rates and the body size of aquatic organisms, or the abundance of primary production (food) in the system—we are still grappling with how to predict coastal ecosystem structure and function under a dynamic reality where all components interact and react to various climate drivers. Audzijonyte and colleagues [1] bring us one step closer to this goal by developing simulations based on a technique broadly known as multispecies size spectrum modelling (MSSS) [2]. They expanded existing versions of this model by incorporating 3 distinct background resource types—pelagic plankton, benthic turf and invertebrates, and macroalgae—and corresponding size spectra, the effects of temperature on marine animal—fish, urchins, and lobsters—life-history processes, and ontogenetic shifts in species diets. By using a fully crossed design, they explored 18 scenarios of benthic and pelagic resource change (increases, decreases or baseline abundance, and slope of size spectra), with and without warming (Fig 1). After forcing each of these scenarios for 150 years, they evaluated changes in standing biomass, fisheries yield, and mean body size of species (or modelled groups) and fish trophic groups.

Audzijonyte and colleagues’ [1] results revealed changes in fish biomass and fisheries yields were more pronounced when background resource levels—planktonic and benthic—shifted (up and downward) by approximately 30%, with or without +2.5°C warming, than when warming occurred without resource shifts. Forcing a synergy between resource abundance and warming often produced little change on the response variables when compared to changes in resource abundance only. Moreover, the magnitude of change was similar between biomass and yield (Figs 3–4 in [1])—at a first glance, this result seems to suggest that the size structure of the modelled groups was not strongly reorganised through the imposed simulated scenarios; however, the mean biomass of many groups also changed in unexpected ways [1], and the authors argued that their simulation results must have been strongly influenced by species interactions. That may also be the reason why the level of change in the evaluated responses was trophic dependent and, at times, nonintuitive. For example, while a 30% decline in plankton resources led to an overall relative decline in biomass (0.61 to 0.86) and yield (0.23 to 0.83; Table H in [1]) across most trophic groups relative to a no-change baseline scenario, an approximately 33% decline in benthic resources led to an increase in biomass (1.11 to 1.23) and yield (1.08 to 1.24) of herbivorous and planktivorous fish. The potential causes of this latter discrepancy were less detailed by the authors but would warrant future exploration given the important role both herbivorous and planktivorous fish play for coastal fisheries [3,4].

The emerging results from changes in the benthic and plankton resources are timely. Benthivores and planktivores were the most affected groups when an approximately 30% decline was imposed on benthic and planktonic resources—specifically, benthivores dropped in biomass by approximately 36% to 38% and planktivores by approximately 30% to 39% depending on whether additional temperature increases were imposed (Table H in [1]). The extent to which benthic primary production fuels energy and nutrient flows across coastal ecosystems remains contentious and is most likely ecosystem and habitat dependent (e.g., [5]). For example, recent evidence suggests that coastal systems, be them temperate rocky reefs [6] or tropical coral reefs [7], are highly subsidised by plankton resources at multiple trophic levels, including benthivores. To a certain extent, the results presented by Audzijonyte and colleagues [1] support that notion by suggesting overall downward yields under reduced plankton resources. Given that current projections of pelagic primary production are alarming [8], future iterations of this novel MSSS may want to explicitly include the physiology and trophic interactions of benthic producers as well as phytoplankton and zooplankton. Audzijonyte and colleagues [1] reinforce the long-recognised (though neglected) dire need to monitor and to understand the dynamics (including abundance, size structure, and physiology) of primary producers—benthic and planktonic—in coastal ecosystems (e.g., [9]), as well as benthic invertebrates.

Multispecies physiologically structured models such as the MSSS are appealing because they can generate a rich number of predictions and emergent outcomes such as size structure, fisheries yield, and ecosystem trophic structure. However, these numerous predictions can be burdened by the level of complexity in the model, making more elaborate MSSS models a far swing from the tractability of simple static theoretical frameworks—in fact, it remains to be seen how the MSSS developed by Audzijonyte and colleagues [1] compares against simpler metabolic theory (e.g., [10]) in terms of predictions and their effects. Such a benchmarking exercise would provide an objective evaluation of the importance of additional MSSS mechanisms—e.g., natural mortality, reproduction, predation, and fishing pressure—in modulating ecosystem structure and functioning. This can help researchers’ decisions on the type of theoretical approach to be employed because an MSSS may demand access to data and parameter information from widely disparate sources, which may not always be available. It is important noting that Audzijonyte and colleagues [1] validated their simulation results by exploring all reasonable combinations of parameter values, benchmarking them against data from a well-established long-term monitoring program. In doing so, the authors offered a roadmap to navigate through simulation-based studies, increasing our capacity to generate credible MSSS—in fact, Audzijonyte and colleagues’ [1] diligent and transparent detailing of their framework in their study is commendable.

Beauty is in eye of the beholder: Although a parameter-rich model might review large sources of uncertainty, it may also uncover future avenues of exploration to further our knowledge. Having stated that, the relevance of any prediction, be it from simple or complex models, is highly contingent on model assumptions and parameter values. Audzijonyte and colleagues [1] explicitly acknowledged that their model aimed “to explore interactions between different climate change processes rather than reflect realistic changes in the ecosystem.” Therefore, future studies designed to empirically validating the simulations of Audzijonyte and colleagues [1] have great potential to advance ecosystem sciences and underpin evidence-based management actions that bolster the resilience of coastal ecosystems.
